# Induction of defence-related proteins by selected plant growth regulators and biocontrol agents against guava root knot nematode, *Meloidogyne enterolobii*

**DOI:** 10.21307/jofnem-2021-081

**Published:** 2021-10-06

**Authors:** N. Ashokkumar, K. Poornima, P. Kalaiarasan, P. Jeyakumar, D. Uma, M. Kavino, S. Dharani, S. Kothai

**Affiliations:** 1 College of Agricultural Sciences, SRM Institute of Science and Technology, SRM Nagar, Kattankulathur, Tamil Nadu, India, 603203; 2 Department of Nematology, Agricultural College and Research Institute, Tamil Nadu Agricultural University, Coimbatore, India; 3 Department of Crop Physiology, Agricultural College and Research Institute, Tamil Nadu Agricultural University, Coimbatore, India; 4 Department of Biochemistry, Agricultural College and Research Institute, Tamil Nadu Agricultural University, Coimbatore, India; 5 Department of Fruit Science, Horticultural College and Research Institute, Tamil Nadu Agricultural University, Coimbatore, India

**Keywords:** Guava, *Meloidogyne enterolobii*, Plant growth regulators, Bioagents

## Abstract

Guava is an important edible and economic fruit crop distributed worldwide. It is widely infested with root knot nematode, *Meloidogyne enterolobii* which plays a vital role in causing economic losses. Several management strategies were performed to enhance the health status of guava and also to reduce root knot nematode infestation. Among the different aspects, application of plant growth regulators on guava plants under nursery conditions against root knot nematode, *M. enterolobii* was performed. The guava plants were treated with Salicylic acid (100 ppm), Jasmonic acid (100 ppm), and Indole 3-Butyric Acid (1000 ppm) alone and in combination of two and three. The result of this study revealed that IBA at 1,000 ppm alone (T3) and combined application of plant growth regulators viz., (T_4_) – Salicylic acid (100 ppm) + Jasmonic acid (100 ppm) + Indole 3-Butyric Acid (1,000 ppm) showed reduction in the nematode population and establishment of new roots (compensatory) and tertiary roots. The combined application of PGRs also increased the Plant height, root length, chlorophyll index, photosynthetic rate, transpiration rate, stomatal conductance, and chlorophyll fluorescence. The activity of various enzymes like total phenols, peroxidase, polyphenol oxidase, acid phosphatase, and phenylalanine ammonia lyase were influenced and developed resistance against root knot nematode, *M. enterolobii.* Under field conditions, application of *Pochonia chlamydosporia* and *Purpureocilium lilacinum* reduced the nematode infestation besides increasing the yield attributes of guava plants.

Guava (*Psidium guajava* L.) is an economically important fruit crop of the Myrtaceae family. It is one of the commercial and important fruits of India widely grown in tropical and sub-tropical regions of the world and hence it is referred to as the ‘poor man’s apple’ and ‘the apple of tropics’ due to the low cost of production and high nutritional value (Radha and Mathew, 2007; Singh, 2005). In Tamil Nadu, Indian farmers have been facing a unique problem in guava trees that showed sudden yellowing followed by bronzing and marginal necrosis of leaves, delayed and poor flowering, shedding of leaves, reduction in fruit size, and decline of guava trees leading to complete destruction of the orchards within a short span of time of one to two years (Ashokkumar and Poornima, 2019). Root-knot nematode infestation at Ayakudi and surrounding villages of Dindigul district which are the major Guava growing area in Tamil Nadu was reported by Poornima et al. (2016) for the first time and the nematode was confirmed to be *Meloidogyne enterolobii* through morphological and molecular means. Studies showed that all the guava cultivars (*Psidium guajava* L.) are susceptible to *M. enterolobii* and also the root exudates of guava cultivars increased the egg hatching ability and decrease the infective juvenile J2 mortality rate and were directly proportional to the time and concentration (Ashokkumar et al., 2019). *M. enterolobii* is regarded as the most aggressive species in comparison to other tropical species of root-knot nematode (Brito et al., 2004) in view of its high reproduction rate, induction of large galls and a very wide host range, and their combinations has become a threat to guava production worldwide leading to the decimation of several guava orchards. Considering the risk of introduction and dissemination of the *M. enterolobii*, it was recently added in European and Mediterranean Plant Protection Organization (EPPO) A2 list (Hallmann and Meressa, 2018). The optimal temperature for growth and development of *M. enterolobii* was 28°C which coincides with the temperature of most of the regions of Tropical Countries especially India and leading to high infestation (Ashokkumar et al., 2019). Various approaches such as physical, cultural, chemical, and biological practices have been used to manage the incidence of root-knot nematode. Cultural practices such as soil solarisation and crop rotation showed limited value in managing nematode infestation due to its broad host range (Ntalli et al., 2010). Hence, there has been an urgency to tackle the destruction caused in guava by this nematode, the aim of the present work was to identify suitable management practices to control the root-knot nematode, *M. enterolobii*.

## Materials and methods

### Nematode isolation and pure culture maintenance

The population of *M. enterolobii* was maintained in Guava (*Psidium guajava*) seedlings under glasshouse conditions. Matured egg masses were collected and kept in distilled water at room temperature (25°C) for two to three days. The total number of hatched second-stage juveniles was counted in four aliquots. The nematode suspension was diluted approximately to about 100 infective juveniles/100g of soil and stored at 4°C for further experiments.

## Preparation of plant growth regulators

### Salicylic acid (100 ppm)

In total, 100 ppm of 1 litre salicylic acid solution was prepared by adding 0.1g of salicylic acid with 500ml of water and few pellets of sodium bicarbonate (to dissolve salicylic acid with water). Final volume of one litre was prepared by adding water to the dissolved solution.

### Jasmonic acid (100 ppm)

Methyl jasmonate solution was prepared by diluting 0.1g of methyl jasmonate in 20ml of ethanol. In total, 980ml of water was added after complete dilution of methyl jasmonate in ethanol and make up to 1 litre of the total solution.

### Indole 3 Butyric acid (1,000 ppm)

In total, 1,000 ppm of 1 litre Indole 3 Butyric acid solution was prepared by weighing 1g of crystalline Indole 3 Butyric acid and dissolved in 20 milliliters of alcohol. The prepared mixture was then added with 980 milliliters of water to make 1,000 ppm of 1 litre Indole 3 Butyric acid solution.

### Mepiquat chloride (500 ppm)

In total, 0.5g of solid Mepiquat chloride was weighed and dissolved in 1,000ml of water to prepare 1 litre of 500 ppm Mepiquat chloride solution.

### Chloromequat chloride (500 ppm)

One litre of 500 ppm Chloromequat chloride solution was prepared by dissolving 0.5g of solid Chloromequat chloride in 1 litre of water.

### Effect of plant growth regulators on *Meloidogyne enterolobii* infected guava plants by pot experiment

The effect of plant growth regulators on the growth of guava seedlings inoculated with root knot nematode, *M. enterolobii* was investigated under greenhouse conditions with three replications for each treatment. Different plant growth regulators viz., Indole 3-Butyric Acid (IBA), Salicylic acid, Jasmonic acid, Mepiquat chloride, Chlormequat chloride were applied alone and in combinations to detect their effect against the root-knot nematode, *Meloidogyne enterolobii* infested guava plants. One month old seedlings were dipped in growth regulators for 3 sec. The treatment details includes T_1_ – Salicylic acid (100 ppm), T_2_ – Jasmonic acid (100 ppm), T_3_ – Indole3-Butyric Acid (1,000 ppm), T_4_ – Salicylic acid + Jasmonic acid + Indole 3-Butyric Acid (100 ppm + 100 ppm + 1,000 ppm), T_5_ – Salicylic acid + Jasmonic acid (100 ppm + 100 ppm), T_6_ – Jasmonic acid + Indole 3-Butyric Acid (100 ppm + 1,000 ppm), T_7_ – Salicylic acid + Indole 3-Butyric Acid (100 ppm + 1,000 ppm), T_8_ – Mepiquat chloride (500 ppm), T_9_ – Chlormequat chloride (500 ppm), and T_10_ – Untreated control. One week after treatment with Plant Growth Regulators, the treated plants were inoculated with infective juveniles at the rate of 1 J_2_/g of soil. The morphological characters Plant height, Root length, Fresh root biomass and Dry root biomass) physiological aspects (Chlorophyll index, Photosynthetic rate, Transpiration rate, Stomatal conductance, and Chlorophyll fluorescence (F_v_/F_m_ ratio), biochemical parameters such as Total phenols, Peroxidase, Polyphenol oxidase, Acid phosphatase, and Phenylalanine ammonia lyase and nematode population in soil and root were recorded at 45, 75, and 105 days after planting (DAP). The effect of such plant growth regulators was evaluated under greenhouse conditions against *M. enterolobii* using guava cultivar ‘Lucknow-49’.

### Methods adopted to determine all physiological parameters and enzyme activities

**Table:** 

Plant height	–	The plant height of guava plants was measured from the ground level to the tip of the most stretched leaf in each replication. The mean values were expressed in cm
Root length	–	The plant was uprooted along with its roots causing lower damage and its length was measured and expressed in cm
Fresh root biomass	–	The roots of guava plants at 45, 75, 105 DAP were uprooted, washed and cleaned to remove the adhering water and soil particles and fresh root weight and was measured and expressed as g plant^-1^
Dry root biomass	–	The roots of guava plants at 45, 75, 105 DAP were uprooted, cleaned and air dried initially followed by oven drying at 65 ± 5^o^C till a constant weight was attained and root weight was measured and expressed as g/plant
Chlorophyll index	–	SPAD meter
Photosynthetic rate	–	Portable photosynthesis system (LI-6400XT, Licor Inc, Nebraska, USA)
Transpiration rate	–	Portable photosynthesis system (LI-6400XT, Licor Inc, Nebraska, USA)
Stomatal conductance	–	Portable photosynthesis system (LI-6400XT, Licor Inc, Nebraska, USA)
Chlorophyll fluorescence	–	Chlorophyll fluorescence meter (Opti-sciences OS5p)
Total phenols	–	Malick and Singh (1980)
Peroxidase		Rad et al. (2007)
Polyphenol oxidase		Mayer et al. (1966)
Acid phosphatase		Dickerson et al. (1984)
Phenylalanine ammonia lyase		Dickerson et al. (1984)

### Effect of different biocontrol agents against root knot nematode, *Meloidogyne enterolobii* under field conditions

The nematode *Meloidogyne enterolobii* infested fields in two different locations: Location-I was laid out at a farmer’s field in Thondamuthur village of Coimbatore district, which was geographically situated in the western Agro-climatic Zone of Tamil Nadu at 11.0250481 N, 76.8839245 E at an altitude of 450m above MSL. Location-II was laid out in a farmer’s field at Avoor village of Thiruvannamalai district, which was geographically situated in the north eastern Agro-climatic Zone of Tamil Nadu at 12.1468353 N, 79.2209262 E at an altitude of 171 m above MSL were selected to test the effect of different biocontrol agents. The treatment parameters included T1 – *Pseudomonas fluorescens* (Pf1) (2.5 kg/ha), T2 – *Bacillus subtilis* (Bs1) (2.5 kg/ha), T3 – *Trichoderma viride* (Tv1) (2.5 kg/ha), T4 – *Pochonia chlamydosporia* (TNAUPc001) (2.5 kg/ha), T5 – *Purpureocillium lilacinum* (TNAUPl001) (2.5 kg/ha), T6 – *Glomus mosseae* (100 g/m^2^), and T7 – Untreated control. The observations were recorded at 30, 60, 90, 120, 150, 180, and 210 days after the application of bio agents. The effect of bio-control agents on root-knot nematode infested guava plants was determined based on different variables viz., Nematode population in soil and root, Morphological characters (Tree height (m), Girth of stem (cm) and Leaf area (cm²)), Nutrient status (Nitrogen content (%), Phosphorous content (%), and Potassium content (%)), Yield characters (Number of fruits per tree, Fruit length (cm), Shoots diameter (cm), Average fruit weight (g), Fruit yield (kg tree-1), and Estimated fruit yield (t/ha)).

### Experimental design

**Table:** 

Variety	:	Luknow-49
Design	:	Randomized block design
Treatments	:	Seven
Replications	:	Ten
Spacing	:	3.0 m × 3.0 m

**Table:** 

		Field trial-I	Field trial-II
Date of planting	:	20.12.2017	12.08.2017
Date of treatment imposed	:	03.01.2018	03.04.2018
Date of harvest	:	04.02.2019	09.01.2019
Soil type	:	Red loamy	Sandy loam

### Statistical analysis

The data generated from various experiments of the present study were analyzed following Gomez and Gomez (1984) method. The package used for analysis was IRRISTAT version 91-1 developed by International Rice Research Institute, Biometrics unit, Philippines.

## Results

### Glasshouse experiment

#### Effect of plant growth regulators on various plant growth parameters in root-knot nematode *M. enterolobii* infested Guava (Lucknow 49)

**Figure 1:**
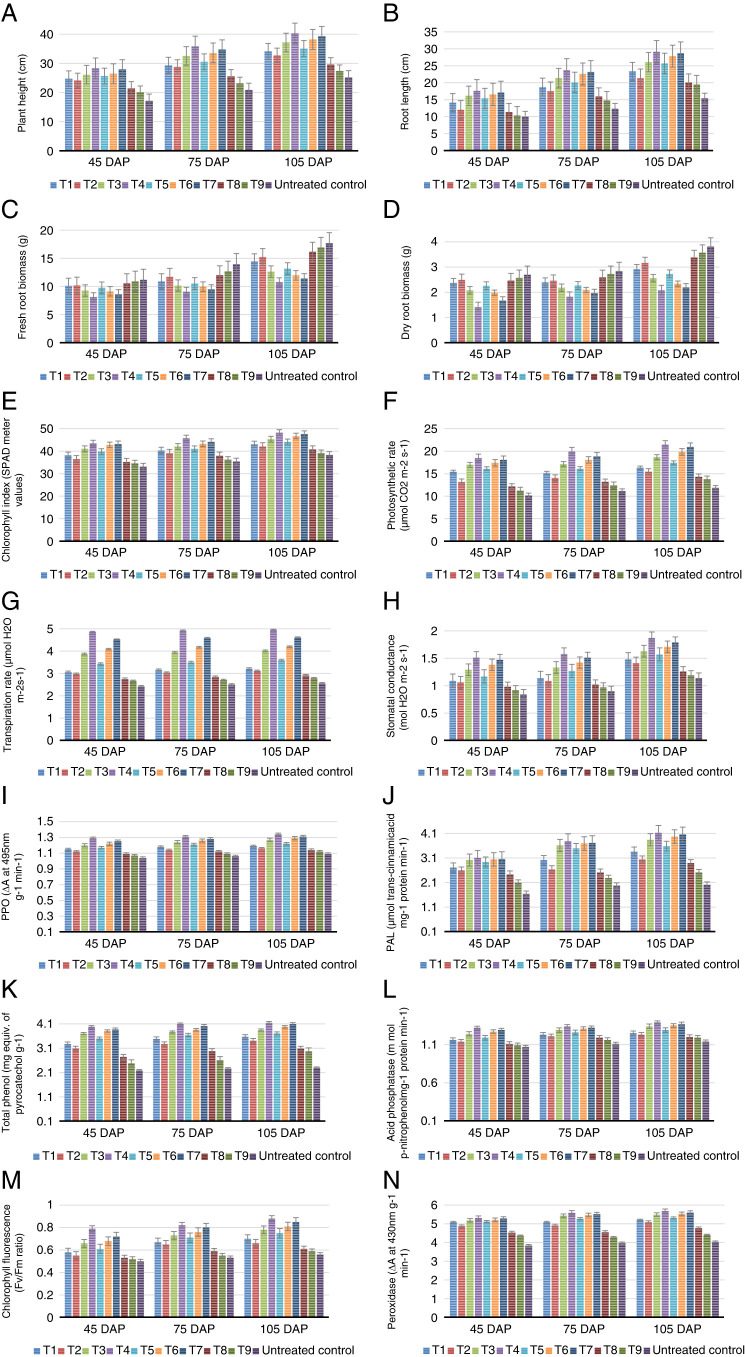
Influence of different plant growth regulators in guava infected by *M. enterolobii* at 45, 75, and 105 days after planting. A. Plant height, B. Root length, C. Fresh root biomass, D. Dry root biomass, E. Chlorophyll index, F. Photosynthetic rate, G. Transpiration rate, H. Stomatal conductance, I. Chlorophyll fluorescence, J. Peroxidase, K. Polyphenol oxidase, L. Phenylalanine ammonia lyase, M. Total phenols, N. Acid phosphatase.

The Lucknow 49 Guava seedlings were treated with plant growth regulators viz., Salicylic acid, Jasmonic acid, Indole 3-Butyric Acid, Mepiquat chloride, Chlormequat chloride alone and in combination by quick dipping method. Morphological, physiological, biochemical, and nematode population measurement parameters of the Guava plants were analyzed at 45, 75, and 105 days after planting. The data presented in Figure 1A–N show that the plant growth regulators significantly influenced the growth (morphological, physiological, and biochemical parameters and showed a reduction in nematode population, egg masses, and female population (*p* ≤ 0.05) (Table 1).

**Table 1.** Effect of plant growth regulators in guava (Lucknow-49) infested with root knot nematode, *M. enterolobii* in soil and root.

					Nematode population in 5g of root								
	Nematode population in 200cc of soil (days)				No of egg masses (days)			No of females (days)			No of eggs/egg masses (days)		
Treatments	0	45	75	105	45	75	105	45	75	105	45	75	105
T_1_ – SA 100ppm	169.33^bc^	167.66^cd^ (0.99)	166.00^cd^ (0.99)	164.66^c^ (0.81)	24.67^c^	23.33^d^ (5.42)	22.00^d^ (5.70)	26.67^bc^	24.66^bc^ (7.53)	24.00^bc^ (2.68)	212.33^bc^	219.66^b^ (**‒**3.45)	206.33^a^ (6.07)
T_2_ – JA 100ppm	176.00^abc^	174.66^abcd^ (0.76)	173.33^bcd^ (0.76)	172.00^bc^ (0.77)	24.67^c^	23.66^d^ (4.08)	22.33^d^ (5.62	28.67^cd^	25.00^c^ (12.79)	24.33^bc^ (2.68)	198.66^a^	204.33^ab^ (**‒**2.85)	218.66^f^ (**‒**7.01)
T_3_ – IBA 1000ppm	181.33^a^	178.66^abc^ (1.47)	176.00^abc^ (1.49)	173.33^abc^ (1.52)	23.33^bc^	21.66^cd^ (7.17)	19.66^c^ (9.23)	26.33^bc^	23.66^bc^ (10.15)	22.66^b^ (4.23)	209.66^abc^	212.33^ab^ (**‒**1.27)	217.33^ef^ (**‒**2.35)
T_4_ – SA100ppm +JA 100ppm + IBA 1000ppm	184.33^a^	180.33^ab^ (2.17)	175.66^abc^ (2.59)	171.00^bc^ (2.65)	20.00^a^	17.66^a^ (11.70)	14.66^a^ (16.99)	22.00^a^	19.66^a^ (10.64)	16.66^a^ (15.26)	214.33^c^	208.00^ab^ (2.95)	206.66^a^ (0.64)
T_5_ – SA 100ppm+JA 100ppm	177.00^abc^	175.33^abcd^ (0.94)	173.66^abcd^ (0.95)	172.33^bc^ (0.77)	24.67^c^	23.33^d^ (5.42)	21.66^d^ (7.16)	26.67^bc^	24.66^bc^ (7.53)	24.33^bc^ (1.34)	200.33^ab^	203.66^ab^ (**‒**1.66)	209.66^bc^ (**‒**2.95)
T_6_ – JA100ppm + IBA1000ppm	181.33^c^	164.33^d^ (1.79)	161.33^d^ (1.83)	158.00^c^ (2.06)	23.00^b^	21.00^bc^ (8.70)	18.66^c^ (11.14)	26.00^b^	23.66^bc^ (9.00)	22.66^b^ (4.23)	219.66^c^	218.66^b^ (0.46)	219.33^f^ (**‒**0.31)
T_7_ – SA100ppm + IBA1000ppm	167.33^abc^	170.33^bcd^ (1.92)	167.00^cd^ (1.96)	163.33^c^ (2.20	21.33^a^	19.00^ab^ (10.94)	16.33^b^ (14.05)	23.33^a^	20.33^a^ (12.87)	18.33^a^ (9.84)	215.33^c^	204.33^ab^ (5.11)	209.66^bc^ (**‒**2.61)
T_8_ – Mepiquat chloride 500 ppm	179.00^ab^	182.66^a^ (**‒**2.04)	186.00^a^ (**‒**1.83	189.33^a^ (**‒**1.79)	26.33^d^	27.33^e^ (**‒**3.78)	28.66^e^ (**‒**4.87)	29.33^de^	28.66^d^ (2.30)	30.66^d^ (**‒**6.98)	207.33^abc^	200.33^a^ (3.38)	208.33^ab^ (**‒**3.99)
T_9_ – Chlormequat chloride 500 ppm	174.66^abc^	179.33^abc^ (**‒**2.67)	183.66^ab^ (**‒**2.41)	187.66^ab^ (**‒**2.18)	27.33^d^	28.33^e^ (**‒**3.65)	29.00^ef^ (**‒**2.36)	31.33^e^	30.00^de^ (4.26)	32.00^d^ (**‒**6.67)	219.66^c^	217.66^b^ (0.91)	219.66^f^ (**‒**0.92)
T_10_ – Untreated control	176.66^abc^	181.66^ab^ (**‒**2.83)	186.00^a^ (**‒**2.39)	190.00^a^ (**‒**2.15)	27.67^d^	29.00^e^ (**‒**4.82)	30.33^f^ (**‒**4.59)	29.67^e^	31.33^e^ (**‒**5.61)	32.33^d^ (**‒**3.19)	218.66^c^	219.66^b^ (**‒**0.46)	212.33^d^ (3.34)

**Notes:** Data are mean of three plants per treatment. Means followed by the same letter do not differ significantly (*p* ≥ 0.05) according to Fisher’s protected LSD test.

### Morphological characters

**Plate 1:**
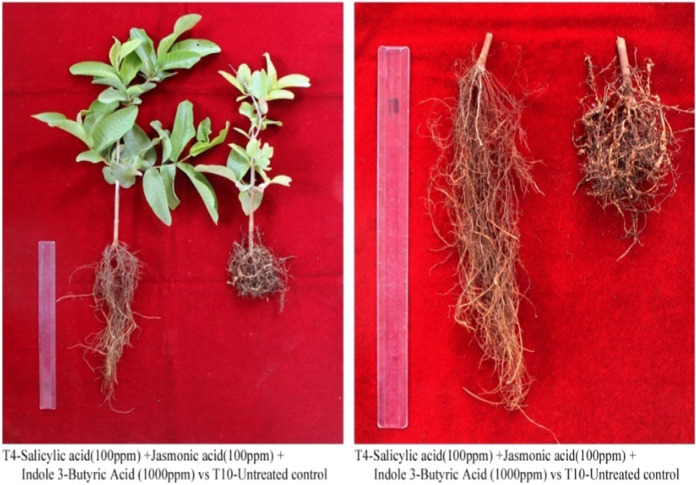
T4 – Salicylic acid (100 ppm) + Jasmonic acid (100 ppm) + Indole 3-Butyric Acid (1,000 ppm) vs T_10_ – Untreated control.

The results showed that plant height measured at 45, 75, and 105 days after planting showed a significant increase in all the treatments and the maximum plant height was recorded at 105 days. The plant height measured for plants treated with two plant growth regulators was higher than the single plant growth regulator treatment. Comparatively, the combination of three plant growth regulators (T4) showed a statistically higher value compared to all treatments and untreated control (T10) (Fig. 1A) (Plate 1). The root length in all the treatments from T1 to T9 was significantly higher than the untreated control. Root length of all the treated and untreated plants increased from 45 to 105 days. The results were statistically significant compared to the untreated control (Fig. 1B). Fresh and dry root biomass of all treatments weighed in 45, 75, and 105 days after planting were statistically and significantly lower than the plants in the untreated control (T10). The fresh root biomass of T10 plants increased from 11.17 g in 45 days to 17.67 in 105 days. Similarly, the dry root biomass of T10 plants increased from 2.69 to 3.81 grams (Fig. 1C, D).

### Physiological parameters

All plant growth regulators used in this study significantly influenced the physiological parameters such as chlorophyll index, photosynthetic rate, transpiration rate, stomatal conductance, and Chlorophyll fluorescence in all treatment and untreated control. The chlorophyll index measured at 105 days was high in T3 compared to T1 and T2. The chlorophyll index was high in T7 compared to T5 and T6 (*p* ≤ 0.05). T4 plants showed higher values when compared to the single and combined plant growth regulator application and the untreated control (Fig. 1E). Plant growth regulators in single and combined applications enhanced the photosynthetic rate of all the treated plants were statistically significant compared to the untreated control. The highest photosynthetic rate was recorded in T4 followed by T7, T6, T3, T5, T1, T2, T8, and T9 (Fig. 1F). The transpiration rate of T4 plants applied with the combination of three plant growth regulators was found to be maximum (4.96, µmol H_2_O m-2 s-1) when compared to other treatments and untreated control (Fig. 1G). Similar results were obtained for stomatal conductance and chlorophyll fluorescence and the highest value was obtained for T4 statistically significant when compared to other treatments and untreated control T10 (Fig. 1H, I).

**Figure:**
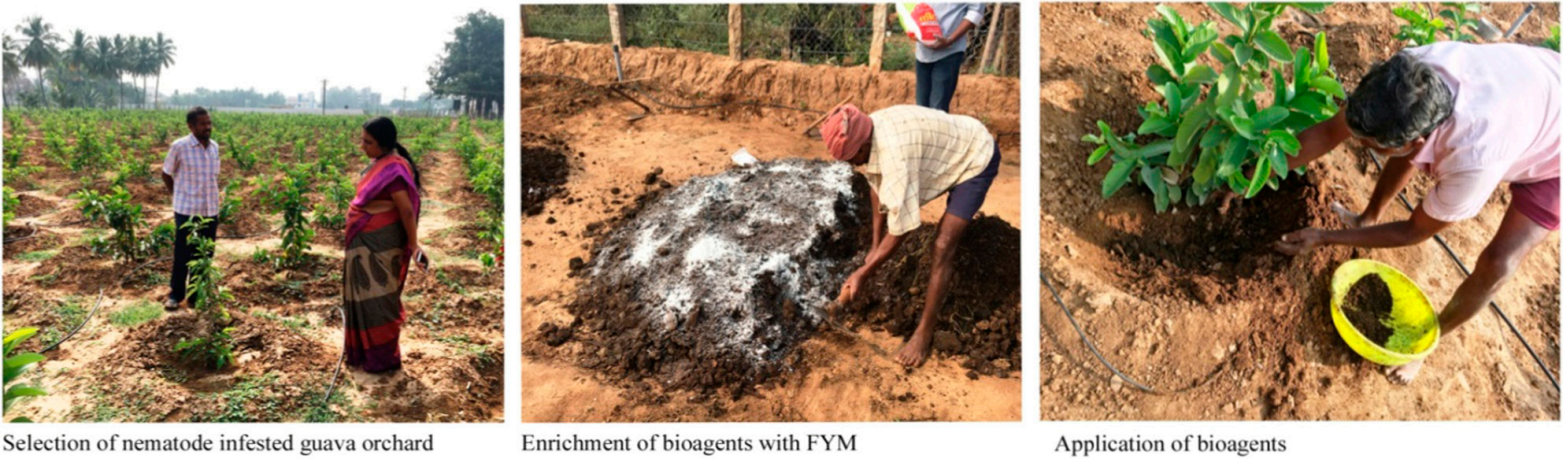


### Biochemical parameters

The induction of peroxidases, polyphenol oxidase, phenylalanine ammonia-lyase, acid phosphatase, and total phenols linking to the defence mechanism of the nematode infected plants treated with plant growth regulators were analyzed (Fig. 1J–N). Treatments from T1 to T7 significantly produced a high percentage of peroxidase compared to T8, T9 and untreated control T10 (Fig. 1J). The analysis of polyphenol oxidase (PPO) activity of plant growth regulator treated plants indicated that the result of T3 plant was higher than T5, similar to T6 and lower than T4 and T7. All the single and combined plant growth regulator treated plants showed higher PPO activity than T8, T9 and untreated control (*p* ≤ 0.05) (Fig. 1K). Among the plants treated with a single plant growth regulator, T3 plants treated with IBA at a concentration of 1,000 ppm showed higher PAL. T3 result was lower than the plants treated with combined plant growth regulators and higher than T8, T9 and untreated control T10 (Fig. 1L). The total phenol of T3 plants was higher than T1, T2 and T5 and lower than T4, T6, and T7. Plants treated with single as well as combined treatments showed higher values for total phenol production (Fig. 1N).

All the biochemical parameters tested were on the raising side when the treated plants were analyzed 45, 75, and 105 days after application of plant growth regulators. The highest value was recorded by T4 followed by T7 and significantly influenced the growth, physiological, and biochemical parameters compared to other treatments and untreated control.

### Nematode population in soil

Treatment with plant growth regulator viz., salicylic acid and Jasmonic acid effectively reduced the nematode population in the soil. A combination of three followed by two plant growth regulators showed high percentages of reduction in nematode population compared to single plant growth promotor application. Reduction of nematode population noted in T3 treated with Indole Butyric Acid at 1,000 ppm concentration was high compared to T1, T2, and untreated control T10. Application of Salicylic acid and Indole butyric acid in combination (T7) effectively reduced the nematode population in soil compared to other plant growth regulator combinations (T5 and T6). The result of the three plant growth regulators (Salicylic acid + Jasmonic Acid + Indole Butyric Acid) combined at a particular concentration specified in this study reduced the nematode population at a high percentage compared to single and combination of two plant growth regulators as well as untreated control. Among T8 and T9, Chlormequat chloride at 500 ppm concentration showed a high percentage of increase in nematode population in the soil in 105 days (Table 1).

### Nematode population in root

Nematode population parameters such as number of females, number of eggs, and egg masses are calculated for 5 gram of root sample collected from Guava plants treated with plant growth regulators in single and in combinations of two and three. A reduction in the number of egg masses was noted from 45th day to 105th day. The highest reduction in egg masses was observed in T3 plants treated with IBA at a concentration of 1,000 ppm. When the plant growth regulators were used in combination (T5, T6, T7), SA + IBA showed a high percentage of reduction in egg mass.

The highest reduction in egg mass was noted in T4 (SA + JA + IBA) combination. The results of T8, T9, and T10 indicated an increase in egg masses. The highest increase was noted in T8 compared to T9 and T10 (Table 1).

Reduction in the female nematode population was noted in T3 in single plant growth regulator treatments and T7 in combined plant growth regulator treatments. Subsequently, an increase in the female population was noted in T8, T9, and T10. The analysis of eggs per egg masses showed that Salicylic acid reduced the number of egg per egg masses, while T2 and T3 resulted in an increased number of eggs/egg mass. The treatments T5, T6, T7, T8, and T9 showed an increase in the number of eggs per egg mass (Table 1).

## Field experiment

### Effect of biocontrol agents in guava (Lucknow-49) infested with root knot nematode, *M. enterolobii* under natural field conditions

All biocontrol agents significantly reduced the nematode population both in soil and plant roots compared to the untreated control. In total, 10 guava plants infested with the root-knot nematode, *M. enterolobii* in each treatment were treated with the Commercial biocontrol agents at regular intervals under natural field conditions in the Experiment. Soil and root samples were collected at 0, 30, 90, 150, and 210 days after the application of biocontrol agents.

### Nematode population in Soil

A high percentage of reduction in nematode population was noted in the plants treated *Pochonia chlamydosporia* (T4) (TNAUPc001) applied at the rate of 2.5kg/ha, in Experiments 1 and 2. Followed by *Purpureocillium lilacinum* (TNAUPl001), *Pseudomonas fluorescens* (Pf1), *Bacillus subtilis* (Bs1), *Trichoderma viride* (Tv1), and *Glomus mosseae* effectively reduced the nematode population in soil compared to the untreated control. However, the lowest nematode population was observed in plants treated with *Pochonia chlamydosporia.* The percentage of reduction in nematode population was 64.91 in 210 days in Experiment I and 76.14 in Experiment II (Table 2).

**Table 2.** Effect of biocontrol agents in guava (Lucknow-49) infested with root knot nematode, *M. enterolobii* in soil.

	Nematode population in 200cc of soil							
Treatments	Initial population	30 days	60 days	90 days	120 days	150 days	180 days	210 days
Experiment-1								
*P. fluorescens* (Pf1)	189.32^b^	180.46^b^ (4.68)	171.24^a^ (9.55)	162.87^c^ (13.97)	151.58^a^ (19.93)	140.32^b^ (25.88)	131.25^b^ (30.67)	123.87^c^ (34.57)
*B.subtilis*(Bs1)	196.45^bc^	190.09bc (3.24)	183.26^b^ (6.71)	172.59^b^ (12.15)	163.34^b^ (16.85)	154.16^d^ (21.53)	140.42^c^ (28.52)	131.52^d^ (33.05)
*T. viride* (Tv1)	177.78^a^	169.21^a^ (4.82)	160.53^a^ (9.70)	155.65^cd^ (12.45)	151.68^a^ (14.68)	145.67^c^ (18.06)	137.67^bc^ (22.56)	122.74^c^ (30.96)
*P. chlamydosporia* (TNAUPc001)	247.54^d^	210.78^d^ (14.85)	188.39^bc^ (23.90)	174.87^e^ (29.36)	153.65^a^ (37.93)	132.82^a^ (46.34)	101.34^a^ (59.06)	86.87^a^ (64.91)
*P. lilacinum* (TNAUPl001	255.25^d^	224.45^e^ (12.07)	190.23^bc^ (25.47)	188.54^d^ (26.14)	169.19^bc^ (33.72)	153.46^d^ (39.88)	129.87^b^ (49.12)	94.48^b^ (62.99)
*G. mosseae*	201.23^c^	193.84^c^ (3.67)	188.41^bc^ (6.37)	179.98^f^ (10.56)	174.34^c^ (13.36)	168.28^e^ (16.37)	163.56^d^ (18.72)	159.29^e^ (20.84)
Untreated control	196.32^bc^	197.87^c^ (0.78)	198.65^c^ (1.17)	199.83^a^ (1.76)	204.25^d^ (3.88)	207.58^f^ (5.42)	210.71^e^ (6.83)	229.45^f^ (14.44)
Experiment-2								
*P. fluorescens* (Pf1)	285.36^b^	262.87^b^ (7.88)	248.54^c^ (12.90)	220.83^c^ (22.61)	199.18^b^ (30.20)	172.37^b^ (39.60)	158.43^c^ (44.48)	141.84^c^ (50.29)
*B.subtilis*(Bs1)	278.48^ab^	257.64^b^ (7.48)	249.25^c^ (10.50)	224.84^c^ (19.26)	201.13^b^ (27.78)	184.23^b^ (33.84)	165.87^d^ (40.44)	150.62^c^ (45.91)
*T. viride* (Tv1)	300.97^c^	289.13c (3.93)	270.24^d^ (10.21)	258.45^d^ (14.13)	237.78^c^ (21.00)	219.55^c^ (27.05)	196.60^e^ (34.68)	173.75^d^ (42.27)
*P. chlamydosporia* (TNAUPc001)	269.35^a^	230.92^a^ (14.27)	200.45^a^ (25.58)	180.06^a^ (33.15)	157.36^a^ (41.58)	132.48^a^ (50.81)	98.09^a^ (63.58)	64.28^a^ (76.14)
*P. lilacinum* (TNAUPl001)	280.42^ab^	259.58^b^ (7.43)	215.26^b^ (23.24)	194.64^b^ (30.59)	165.92^a^ (40.83)	140.47^a^ (49.91)	112.64^b^ (59.83)	79.85^b^ (71.52)
*G. mosseae*	300.97^d^	289.13^d^ (3.93)	270.24^e^ (10.21)	258.45^d^ (14.13)	237.78^c^ (21.00)	219.55^c^ (27.05)	196.60^f^ (34.68)	173.75^e^ (42.27)
Untreated control	278.97^ab^	285.52^c^ (2.29)	290.48^e^ (3.96)	296.31^e^ (5.85)	298.52^d^ (6.55)	301.26^d^ (07.40)	305.95^g^ (8.82)	308.72^f^ (9.64)

**Notes:** Data are mean of 10 plants per treatment, obtained from 2018 and 2019 experiments. Means followed by the same letter do not differ significantly (*p* ≥ 0.05) according to Fisher’s protected LSD test.

### Nematode population in roots

The analysis of the plant roots in 0, 30, 90, 150, and 210 days after the application of biocontrol agents showed that *Pochonia chlamydosporia* recorded the lowest egg mass of 2.85 per 5 gram of the root, analyzed in 210 days in both Experiments I and II. The results did not differ significantly within T4 and T5 and between T2, T3, and T4 in Experiment I. The results obtained in Experiment II revealed that the plants of T4 and T5 treated with *Pochonia chlamydosporia* and *Purpureocillium lilacinum* differed significantly.

The untreated control plants showed a increase trend for the same parameters tested, i.e. the number of egg masses increased from 16.94 to 23.48 egg masses/5g of the root, similarly increase in female population from 29.26 to 36.42 females was observed. The number of eggs per egg mass increased from 158.23 to 175.53 in Experiments I and II (Table 3).

**Table 3.** Effect of biocontrol agents in guava (Lucknow-49) infested with root knot nematode, *M. enterolobii* in root.

	Nematode population in roots (5g)														
	No of egg masses (days)					No of females (days)					No of eggs/egg masses (days)				
Treatments	00	30	90	150	210	00	30	90	150	210	00	30	90	150	210
Experiment-1															
*P. fluorescens* (Pf1)	17.32^b^	13.45^a^ (22.34)	11.89^c^ (31.35)	09.83^c^ (43.24)	07.39^b^ (57.33)	30.48^a^	22.23^b^ (27.07)	19.23^c^ (36.91)	16.52^c^ (45.80)	13.66^b^ (55.18)	149.98^a^	145.65^a^ (2.89)	141.42^a^ (5.71)	138.43^b^ (7.70)	135.32^b^ (9.77)
*B.subtilis*(Bs1)	19.54^d^	15.68^d^ (19.75)	12.32^cd^ (36.95)	10.42^c^ (46.67)	09.26^c^ (52.61)	29.19^a^	25.12^c^ (13.94)	22.74^d^ (22.10)	20.34^d^ (30.32)	17.51^c^ (40.01)	172.38^d^	160.54^c^ (6.87)	154.57^bc^ (10.33)	151.63^c^ (12.04)	148.07^c^ (14.10)
*T. viride* (Tv1)	16.38^a^	14.56^b^ (11.11)	12.63^d^ (22.89)	10.35^c^ (36.81)	09.34^c^ (42.98)	34.52^c^	30.09^d^ (12.83)	25.82^e^ (25.20)	21.84^e^ (36.73)	18.48^d^ (46.47)	163.34^bc^	160.94^c^ (1.47)	157.32^c^ (3.69)	154.81^c^ (5.22)	152.29^c^ (6.77)
*P. chlamydosporia* (TNAUPc001)	18.37^c^	14.84^bc^ (19.22)	10.95^b^ (40.39)	04.64^a^ (74.74)	02.86^a^ (84.43)	34.87^c^	21.32^ab^ (38.86)	14.09^b^ (59.59)	07.56^a^ (78.32)	03.98^a^ (88.59)	163.87^bc^	152.42^b^ (6.99)	140.75^a^ (14.11)	129.43^a^ (21.02)	115.35^a^ (29.61)
*P. lilacinum* (TNAUPl001)	19.65^d^	15.26^cd^ (22.34)	10.12^a^ (48.50)	05.64^b^ (71.30)	02.85^a^ (85.50)	32.35^b^	20.54^a^ (36.51)	12.45^a^ (61.51)	09.39^b^ (70.97)	04.54^a^ (85.97)	159.56^b^	151.5^ab^ 8(5.00)	145.39^ab^ (8.88)	136.24^ab^ (14.62)	129.48^b^ (18.85)
*G. mosseae*	20.46^e^	18.23^e^ (10.90)	16.43^e^ (19.70)	14.65^d^ (28.40)	12.62^d^ (38.32)	37.98^d^	32.94^e^ (13.27)	30.64^f^ (19.33)	26.98^f^ (28.96)	23.76^e^ (37.44)	167.67^cd^	162.32^c^ (3.19)	158.24^cd^ (5.62)	155.85^c^ (7.05)	153.68^c^ (8.34)
Untreated control	16.94^ab^	19.56^f^ (13.39)	21.65^f^ (21.76)	22.32^e^ (24.10)	23.48^e^ (27.85)	29.26^a^	30.29^d^ (3.40)	32.38^g^ (9.64)	35.63^g^ (17.88)	36.42^f^ (19.66)	158.23^b^	164.09^c^ (3.57)	167.83^d^ (5.72)	171.34^d^ (7.65)	175.53^d^ (9.86)
Experiment-2															
*P. fluorescens* (Pf1)	21.24^bc^	16.20^b^ (23.73)	12.57^b^ (40.82)	08.90^b^ (58.10)	06.41^b^ (69.82)	30.38^ab^	22.08^ab^ (27.32)	19.81^b^ (34.79)	14.61^c^ (51.91)	10.64^c^ (64.98)	163.42^ab^	155.45^ab^ (4.88)	151.73^b^ (7.15)	144.24^b^ (11.74)	135.54^b^ (17.06)
*B.subtilis*(Bs1)	22.89^e^	18.34^c^ (19.88)	15.78^d^ (31.06)	12.06^c^ (47.31)	07.59^c^ (66.84)	36.87^d^	28.46^d^ (22.81)	20.24^bc^ (45.10)	16.35^d^ (55.66)	11.73^d^ (68.19)	172.76^c^	163.31^cd^ (5.47)	152.85^b^ (11.52)	139.68^b^ (19.15)	132.36^b^ (23.39)
*T. viride* (Tv1)	20.45^a^	17.81^c^ (12.91)	13.69^c^ (33.06)	11.51^c^ (43.72)	08.34^d^ (59.22)	30.37^ab^	24.34^c^ (19.86)	20.92^c^ (31.12)	16.09^d^ (47.02)	12.44^e^ (59.04)	165.62^bc^	161.45^bc^ (2.52)	156.43^b^ (5.55)	150.53^c^ (9.11)	147.62^c^ (10.87)
*P. chlamydosporia* (TNAUPc001)	21.68^cd^	14.50^a^ (33.12)	10.80^a^ (50.18)	05.84^a^ (73.06)	02.57^a^ (88.15)	29.35^a^	20.78^a^ (29.20)	14.28^a^ (51.35)	09.52^a^ (67.56)	04.94^a^ (83.17)	157.47^a^	149.39^a^ (5.13)	138.65^a^ (11.95)	129.04^a^ (18.05)	123.50^a^ (21.57)
*P. lilacinum* (TNAUPl001)	22.24^d^	16.20^b^ (27.16)	13.57^c^ (38.98)	08.90^b^ (59.98)	03.41^c^ (84.66)	31.38^bc^	23.08^bc^ (26.45)	19.81^b^ (36.87)	10.61^b^ (66.19)	08.64^b^ (72.47)	167.42^bc^	162.45^cd^ (2.97)	154.73^b^ (7.58)	142.24^b^ (15.04)	131.54^b^ (21.43)
*G. mosseae*	20.89^ab^	19.34^d^ (7.42)	16.78^e^ (19.67)	13.06^d^ (37.48)	11.59^e^ (44.52)	31.87c	28.46^d^ (10.70)	25.24^d^ (20.80)	22.35^e^ (29.87)	19.73^f^ (38.09)	170.78^bc^	168.78^de^ (1.17)	165.85^c^ (2.89)	161.68^d^ (5.33)	155.36^d^ (9.03)
Untreated control	23.72^f^	26.43^e^ (10.25)	29.26^f^ (18.93)	32.02^e^ (25.92)	34.56^f^ (31.37)	35.46^d^	37.82^e^ (6.24)	40.24^e^ (11.88)	43.52^f^ (18.52)	45.68^g^ (22.37)	171.76^c^	172.31^e^ (0.32)	176.54^d^ (2.71)	178.34^e^ (3.69)	179.54^e^ (4.33)

**Notes:** Data are mean of 10 plants per treatment, obtained from 2018 and 2019 experiments. Means followed by the same letter do not differ significantly (*p* ≥ 0.05) according to Fisher’s protected LSD test.

### Effect of biocontrol agents on various plant growth parameters and induction of resistance in root-knot nematode *M. enterolobii* infested guava plants under natural field conditions

**Plate 2:**
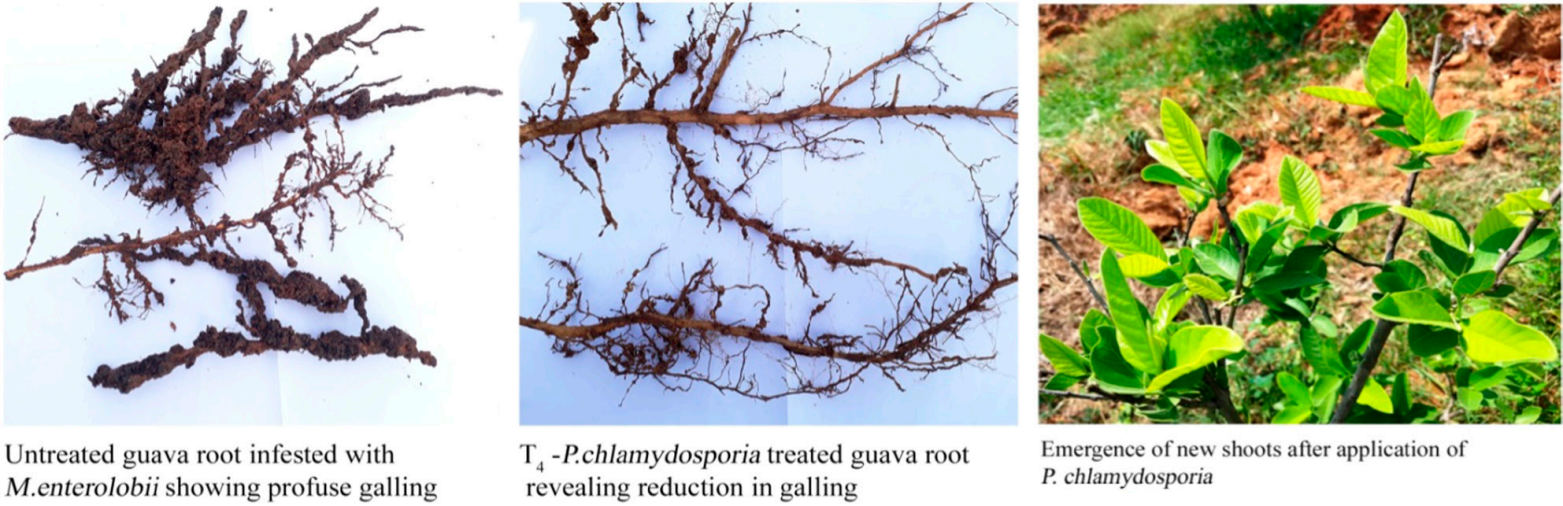
Field observations on application of different biocontrol agents.

All the plants treated with commercial biocontrol agents at regular intervals during the year 2018 significantly enhanced the plant growth and yield of guava plants under natural field conditions. Application of *Pochonia chlamydosporia* (T4) (Plate 2) and *Purpureocilium lilacinum* (T5) enhanced the tree height girth of the stem and leaf area in both vegetative and fruiting stages of nematode infested guava plants chosen in Experiments I and II. *Pseudomonas fluorescens* (Pf1) *Bacillus subtilis* (Bs1) showed statistically similar results. Comparatively all the biocontrol agents used in this study have improved both vegetative and fruiting stage parameters of guava plants. *Glomus mosseae* was found to be least effective in both Experiments I and II (Table 4).

**Table 4.** Effect of biocontrol agents on Plant growth parameters of guava cv. Lucknow-49 challenged with root knot nematode, *M. enterolobii*.

	Yield parameters											
	At vegetative stage			At fruiting stage								
Treatments	Tree height (m)	Girth of stem (cm)	Leaf area (cm^2^)	Tree height (m)	Girth of stem (cm)	Leaf area (cm^2^)	No. of fruits/tree (nos)	Fruit length (cm)	Shoots diameter (cm)	Average fruit weight (g)	Fruit yield/tree (kg tree^**‒**1^)	Fruit yield t/ha
Experiment-1												
*P. fluorescens* (Pf1)	1.36^b^	12.29^b^	74.12^b^	1.74^b^	12.41^b^	74.24^b^	40.00^b^	7.41^b^	8.22^bc^	278.12^b^	11.12^c^	10.91^c^
*B. subtilis*(Bs1)	1.34^bc^	12.00^b^	72.67^bc^	1.72^b^	12.14^b^	72.77^b^	39.60^b^	7.30^bc^	8.09^cd^	264.00^bc^	10.45^d^	10.25^d^
*T. viride* (Tv1)	1.29^cd^	11.42^c^	70.00^c^	1.68^bc^	11.56^c^	70.11^bc^	38.40^bc^	7.11^bc^	7.93^d^	259.00^c^	9.95^e^	9.76^e^
*P. chlamydosporia* (TNAUPc001)	1.80^a^	16.00^a^	82.10^a^	2.14^a^	16.19^a^	82.24^a^	45.20^a^	8.68^a^	8.59^a^	336.20^a^	15.20^a^	14.91^a^
*P. lilacinum* (TNAUPl001	1.78^a^	15.91^a^	79.27^a^	2.11^a^	16.04^a^	79.39^a^	43.60^a^	8.61^a^	8.47^ab^	321.10^a^	14.00^b^	13.73^b^
*G. mosseae*	1.24^de^	11.26^cd^	66.21^d^	1.59^c^	11.40^cd^	66.32^cd^	37.10^c^	6.89^c^	7.62^e^	231.00^d^	8.57^f^	8.41^f^
Untreated control	1.19^e^	10.84^d^	64.00^d^	1.43^d^	11.00^d^	64.10^d^	34.10^d^	6.20^d^	7.36^e^	165.00^e^	5.63g	5.52^g^
Experiment-2												
*P. fluorescens* (Pf1)	1.44^b^	12.38^b^	75.31^b^	1.89^c^	12.45^b^	75.54^b^	37.16^c^	7.36^b^	8.44^ab^	261.06^b^	9.70^c^	9.52^c^
*B. subtilis*(Bs1)	1.39^bc^	12.17^bc^	74.70^b^	1.83^c^	12.24^bc^	75.09^b^	34.37^d^	7.31^bc^	8.35^b^	239.14^c^	8.22^d^	8.06^d^
*T. viride* (Tv1)	1.32^cd^	11.61^cd^	68.84^c^	1.71^d^	11.67^cd^	70.05^c^	32.04^e^	7.08^c^	8.31^bc^	259.00^b^	8.30^d^	8.14^d^
*P. chlamydosporia* (TNAUPc001)	1.86^a^	16.18^a^	82.07^a^	2.49^a^	16.29^a^	82.25^a^	43.02^a^	8.43^a^	8.69^a^	310.40^a^	13.35^a^	13.10^a^
*P. lilacinum* (TNAUPl001	1.81^a^	16.02^a^	80.89^a^	2.28^b^	16.17^a^	81.16^a^	41.01^b^	8.39^a^	8.63^a^	304.20^a^	12.48^b^	12.24^b^
*G. mosseae*	1.28^de^	11.39^d^	63.13^d^	1.60^e^	11.44^d^	65.17^d^	30.03^f^	6.72^d^	8.29^c^	220.03^d^	6.61^e^	6.48^e^
Untreated control	1.21^e^	11.02^d^	62.18^d^	1.42^f^	11.09^d^	63.09^d^	26.11^g^	6.04^e^	8.23^d^	149.01^e^	3.89^f^	3.82^f^

**Notes:** Data are mean of 10 replicated plants per treatment, obtained from 2018 and 2019 experiments. Means followed by the same letter do not differ significantly (*p* ≥ 0.05) according to Fisher’s protected LSD test.

Further, this study showed that treating the *M. enterolobii* infested guava plants with biocontrol agent *Pochonia chlamydosporia* reduced the nematode population and showed greater improvement on plant growth and yield parameters. Next to *Pochonia chlamydosporia*, *P. lillacinum* also showed a greater impact in reducing the nematode population by increasing the plant growth and yield parameters.

## Discussion

Current study results showed that the application of plant growth regulators in combination increased plant growth parameters of Lucknow-49, Guava plants and resulted in producing enormous new root proliferation and reduced the nematode infection. Among different treatments, the use of combined plant growth regulators such as Salicylic acid (100 ppm) + Jasmonic acid (100 ppm) + Indole 3-Butyric Acid (1,000 ppm) showed a positive influence on plant height, root length, root number, and promoted the formation of the tertiary roots. Previous studies on these plant growth regulators such as Salicylic acid and Jasmonic acid-enhanced plant resistance against plant parasitic-nematodes (Charehgani et al., 2014; Oka et al., 1999). Salicylic acid played its role in enhancing the physiological plant defence responses against plant pathogens through systemic acquired resistance (Molinari and Baser, 2010).

Results from glasshouse showed that the application of salicylic acid, Jasmonic acid and Indole 3-Butyric Acid at specific combination increased the activity of total phenols, peroxidase, polyphenol oxidase, acid phosphatase, and phenylalanine ammonia-lyase at different intervals. Such an increase in biochemical compounds enhanced the development of resistance against root-knot nematodes. The rise in biochemical compounds may be due to the rapid breakdown of bound phenols leading to the formation of lignin that offers resistance to nematodes. The enzyme peroxidase plays a significant role in the defence mechanism, catalyzes the process of condensation phenols into lignin leading to a hypersensitive reaction and an activated resistance against the invading pathogen (Dignum et al., 2001). In general, an increase in peroxidase activity at the later stages of the infection process activates the free radicals that inhibit the activities of the pathogen (Hammerschmidt et al., 1982).

Acid phosphatase is an enzyme closely linked with the Mi gene, confers resistance against root-knot nematodes (Williamson and Colwell, 1991). It catalyzes the process of hydrolysis of inorganic phosphates from phosphomonoesters at reduced pH level and offers resistance to plants against nematodes. Increased Phenylalanine ammonia-lyase enzyme induces the activation of defence compound, trans-cinnamic acid and initiates the lignin biosynthetic pathway involved in the resistance against nematodes (Brueske, 1980).

Soil is home to still unknown organisms with antagonistic potential (Sikora et al., 2003). Many of these organisms are responsible for the reduced nematode infestation (Sikora et al., 2003). Based on this concept, several biocontrol agents were developed and used as root-knot nematode controlling agents. For example, the egg-parasitic fungi *Pochonia chlamydosporia* and *Purpureocillium lilacinum* were the most common and effective biological agents targeting the root-knot nematodes (Anastasiades et al., 2008; Atkins et al., 2003; Carneiro et al., 2011; Hidalgo-Diaz et al., 2000; Khan et al., 2006; Moosavi et al., 2010). Till now, *P. lilacinus* was found to be one of the most promising biological fungi to control plant-parasitic nematode populations beneath numerous conditions (Kiewnick and Sikora, 2006). In general, *P. lilacinum* strains were found to be less active than *P. chlamydosporia* strains in reducing the hatching ability of the nematode eggs (Chen and Chen, 2003). The present study also confirmed that *Pochonia chlamydosporia* offers effective nematode control over various growth stages of guava under field conditions. Similarly, several other studies reported *P. chlamydosporia* as one of the most promising effective bio-control agents against root-knot nematodes under field conditions (Viaene and Abawi, 2000).

## Conclusion

In India, Lucknow 49 Guava is a popular and widely cultivated variety grown as a commercial crop. It is highly susceptible to nematode infestation and causing an economic loss at nurseries and in the established orchards of Tamil Nadu. Management practices are insignificant to control the nematodes. The nematode *M. enterolobii* infestation in Guava seedlings at the nursery stage can be managed through the application of plant growth regulators at a specific combination used in this study. The active ingredients of three plant growth regulators Salicylic acid, Jasmonic acid, and Indole 3-Butyric Acid might have worked synergically and enhanced the morphological, physiological, and biochemical parameters of the nematode infested guava plant Lucknow 49. Induction of defence-related proteins might have offered protection against the nematode infestation in addition to the plant growth regulators used in a combination of three. The combined effect of the plant growth regulators reduced the female nematode population and egg masses in both soil and in roots of the infested guava plants of this study.

For the standing Guava crop, the application of *Pochonia chlamydosporia* and *Purpureocilium lilacinum* enhanced the tree height girth of the stem and leaf area in both vegetative and fruiting stages of nematode infested guava plants chosen for this study. The lowest nematode population was observed in plants treated with *Pochonia chlamydosporia*. The guava plant treated with *Pochonia chlamydosporia* recorded the lowest female nematode population and egg mass in plant root compared to all the biocontrol agents used in this study. Comparatively all the biocontrol agents used in this study have improved both vegetative and fruiting stage parameters of guava plants. *Glomus mosseae* was found to be least effective in both Experiments I and II.

Further, this study suggests that treating the guava seedlings with plant growth regulators before transplantation reduced the *M. enterolobii* infestation by improving the emergence of secondary and tertiary roots (compensatory roots) to withstand the nematode infestation at nursery stage. Such treatments also enhance the activity of defence enzymes present within the plants against the nematodes and resistance induction. In standing crop, the application of biocontrol agents regularly at monthly intervals prevent the nematode infestation and reduce the nematode populations already present within the plant roots and in the soil. This study suggests quick dipping of guava seedlings with IBA + SA + JA (Enhance compensatory roots and defence enzymes) and transplanting in the main field by initial application of *Pochonia chlamydosporia* or *Purpureocillium lilacinum* will be an effective management practice to control root knot nematode, *M. enterolobii*.
